# Pitfalls on sample preparation for *ex vivo* imaging of resected cancer tissue using enzyme-activatable fluorescent probes

**DOI:** 10.18632/oncotarget.26320

**Published:** 2018-11-13

**Authors:** Ai Mochida, Fusa Ogata, Yasuhiro Maruoka, Tadanobu Nagaya, Ryuhei Okada, Fuyuki Inagaki, Daiki Fujimura, Peter L. Choyke, Hisataka Kobayashi

**Affiliations:** ^1^ Molecular Imaging Program, Center for Cancer Research, National Cancer Institute, National Institutes of Health, Bethesda, Maryland, United States of America

**Keywords:** fluorescence-guided surgery, activatable probe, enzyme, *ex vivo* imaging, temperature

## Abstract

*In vivo* and *ex vivo* fluorescence imaging-assisted surgery can aid in determining the margins of tumors during surgical resection. While a variety of fluorescent probes have been proposed for this task, small molecule enzyme-activatable fluorescent probes are ideal for this application. They are quickly activated at tumor sites and result in bright signal with little background, resulting in high sensitivity. Testing in resected specimens, however, can be difficult. Enzymes are usually stable after freezing and thawing but catalytic reactions are generally temperature-dependent. Therefore, tissue sample temperature should be carefully considered. In this study two enzyme activatable probes, γ-glutamylhydroxymethyl rhodamine green (gGlu-HMRG) that reacted with γ-glutamyltransferase and SPiDER-βGal that reacted with β-galactosidase, were employed to determine the effects of temperature on fluorescence signal kinetics in both fresh and frozen and then thawed *ex vivo* experimental ovarian cancer tissue samples. The results suggest γ-glutamyltransferase was less sensitive to temperature than β-galactosidase. Fresh samples showed higher fluorescence signals of gGlu-HMRG compared with thawed samples likely because the freeze-thaw cycle decreased the rate of internalization of the activated probe into the lysosome. In contrast, no significant difference of SPiDER-βGal fluorescence signal was observed between fresh and frozen tissues. In conclusion, although imaging of fresh samples at 37°C is the best condition for both probes, successful imaging with gGlu-HMRG could be achieved even at room temperature with thawed samples. We demonstrate that temperature regulation and tissue handling of resected tissue are two pitfalls that may influence *ex vivo* imaging signals with enzyme-activatable fluorescent probes.

## INTRODUCTION

Intraoperative optical fluorescence molecular imaging provides real-time image guidance to surgeons to identify precise tumor margins and detect tiny tumor foci both of which result in improved resections with less residual disease, decreasing the risk of recurrence [1]. Fluorescence imaging is low-cost and portable, yet highly sensitive and minimally invasive and does not utilize ionizing radiation [2–5]. Conventional imaging probes, called “always-on” probes, continuously emit signals similar to conventional contrast agents used in computed tomography (CT), magnetic resonance imaging (MRI), and angiography and the images depend on the biodistribution of the probe, resulting in relatively high background signals and requiring time to wash out the background signal from the body. In order to overcome this limitation, “activatable” probes have been introduced. They are turned on only after binding to a specific target found on tumors and, as a result, maximize the target signal while minimizing the background signal, resulting in a high target to background ratio (TBR) [6–8].

**Figure 1:**
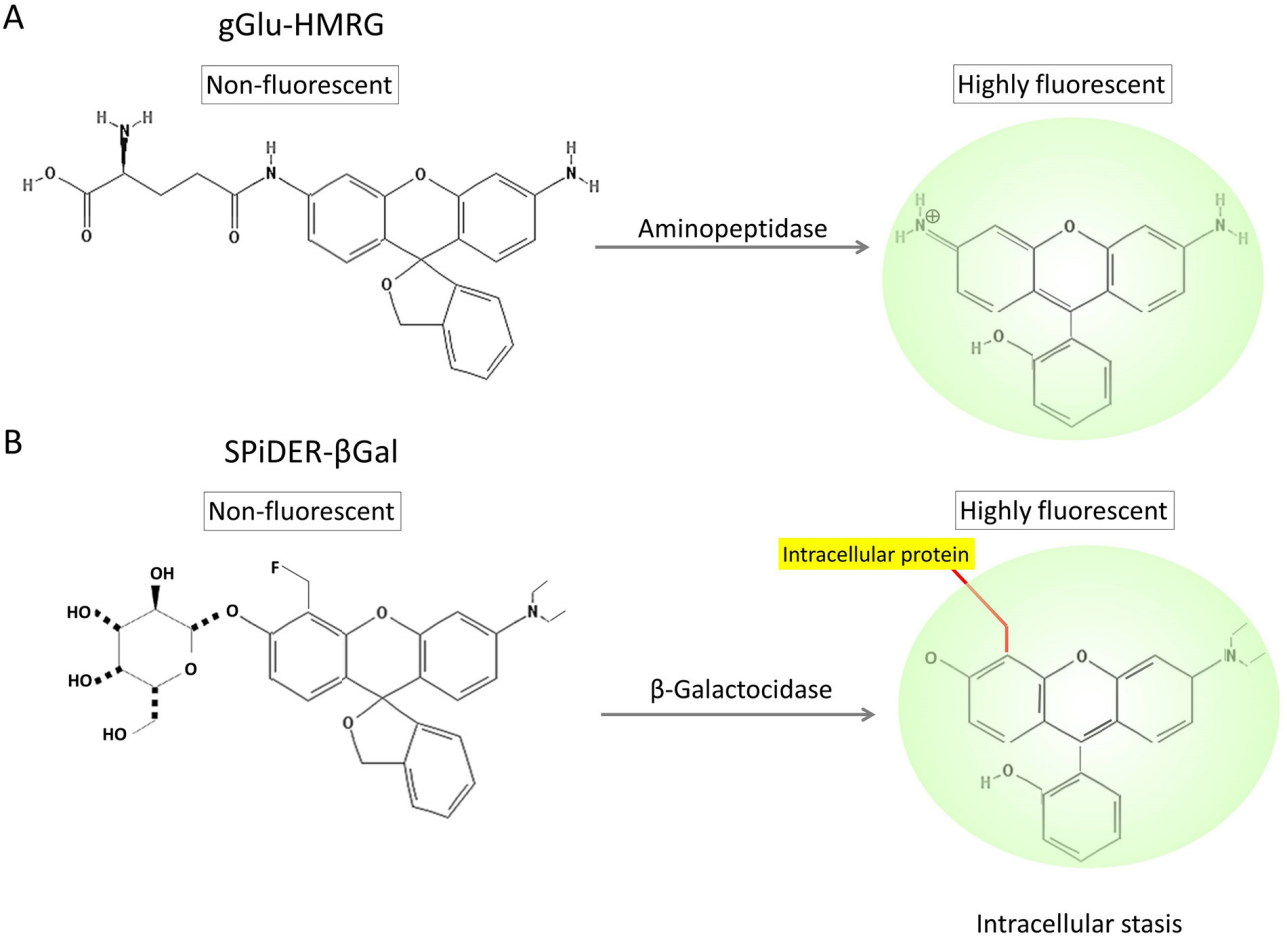
Chemical structures of gGlu-HMRG **(A)** and SPiDER-βGal **(B)**, and their enzymatic reaction with aminopeptidase and β-galactosidase.

Small molecule enzyme-activatable fluorescence probes are rapidly cleaved and activated by a single enzymatic reaction using targeted endopeptidases. Often this process occurs in less than an hour making it practical for clinical translation [9, 10]. Activatable probes have been investigated in preclinical animal models and some of them have recently proceeded to clinical trials [11–13]. Such probes can target cancer-associated enzymes including cathepsin-B, cathepsin-L [14, 15], matrix metalloproteinases-2 (MMP-2) [16, 17], β-galactosidase (β-Gal) [18–20] and γ-glutamyltransferase (GGT) (Figure 1) [9, 21, 22]. In some cases small molecule enzyme-activatable probes can be applied topically during surgery and can be rapidly activated, thus causing minimal disruption to workflow.

Determining the positivity of tumor margins is often important to achieve optimal surgical results. Thus, during surgery, intraoperative frozen section analysis (IFSA) is frequently performed to assess resection margins. However, IFSA is a labor-intensive technique and requires both cost and time during which the patient remains under anesthesia [23, 24]. IFSA can add more than 30 min to a surgical procedure [25, 26]. Moreover, it can be limited by artifacts and undersampling, increasing the possibility of false negative results [23, 24]. In comparison, the topical application of a rapidly acting enzyme-reactive probe to extracted specimens could provide a rapid and easy assessment of tumor margin.

β-Gal and GGT are overexpressed in a variety of cancers, and are potential candidates for intraoperative enzyme activated probes. In a previous report, we described the fluorescence signal and the kinetics generated by HMRef-βGal and SPiDER-βGal both activated by β-galactosidase and gGlu-HMRG which is activated by GGT in ovarian cancer cell lines at 37°C [10]. HMRef-βGal, the original probe [19], behaved with similar kinetics to gGlu-HMRG probe at this temperature although the fluorescence signal of HMRef-βGal was much lower than that of gGlu-HMRG. This led to the selection of SPiDER-βGal as the preferred β-galactosidase-activated probe. However, in reality such probes must function at temperatures less than 37°C and their performance under other temperature conditions is poorly understood.

In this study, we compared incubation temperature and tumor tissue handling (fresh or frozen then thawed) for probes targeting two enzymes, GGT and β-galactosidase. Specimen temperature could affect the ability to see tumor margins. Therefore, we investigated fluorescence signal kinetics of gGlu-HMRG (Figure 1A) and SPiDER-βGal (Figure 1B) at different temperatures in *in vitro* and *ex vivo* imaging using cancer cell lines expressing the target enzyme.

## RESULTS

### *In vitro* fluorescence imaging

**Figure 2:**
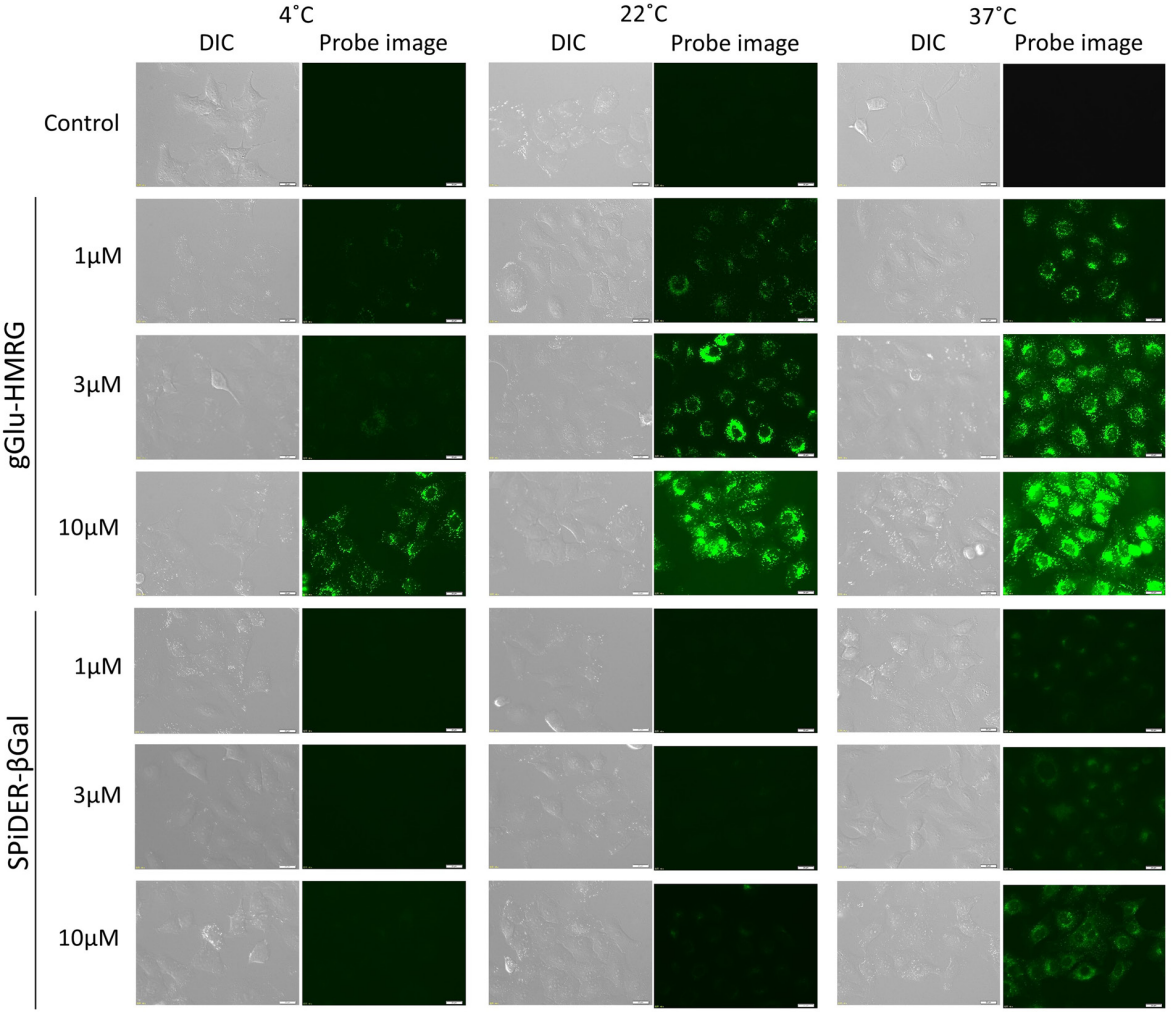
Fluorescence microscopic studies SHIN3 cells were incubated with 1μM, 3μM, and 10μM gGlu-HMRG or SPiDER-βGal for 10 min at 4°C, 22°C, and 37°C. Camera setting: excitation wavelength range is 450 – 490 nm and emission wavelength range is 500 – 550 nm. Exposure time: 1000ms. Bars are 20 μm.

SHIN3 cells were serially observed under fluorescence microscopy up to 30 minutes after the addition of gGlu-HMRG or SPiDER-βGal. When the concentration of the probe or the temperature was higher, the fluorescence signals activated in cells were stronger (Figure 2). For cells incubated with 10 μM of gGlu-HMRG, intracellular accumulation of the activated probes could be seen even at 4°C. The florescence signal of SPiDER-βGal was significantly lower at 4°C and 22°C than that at 37°C, suggesting that SPiDER-βGal was more sensitive to temperature than gGlu-HMRG.

### Persistence of fluorescence signal *in vitro*

**Figure 3:**
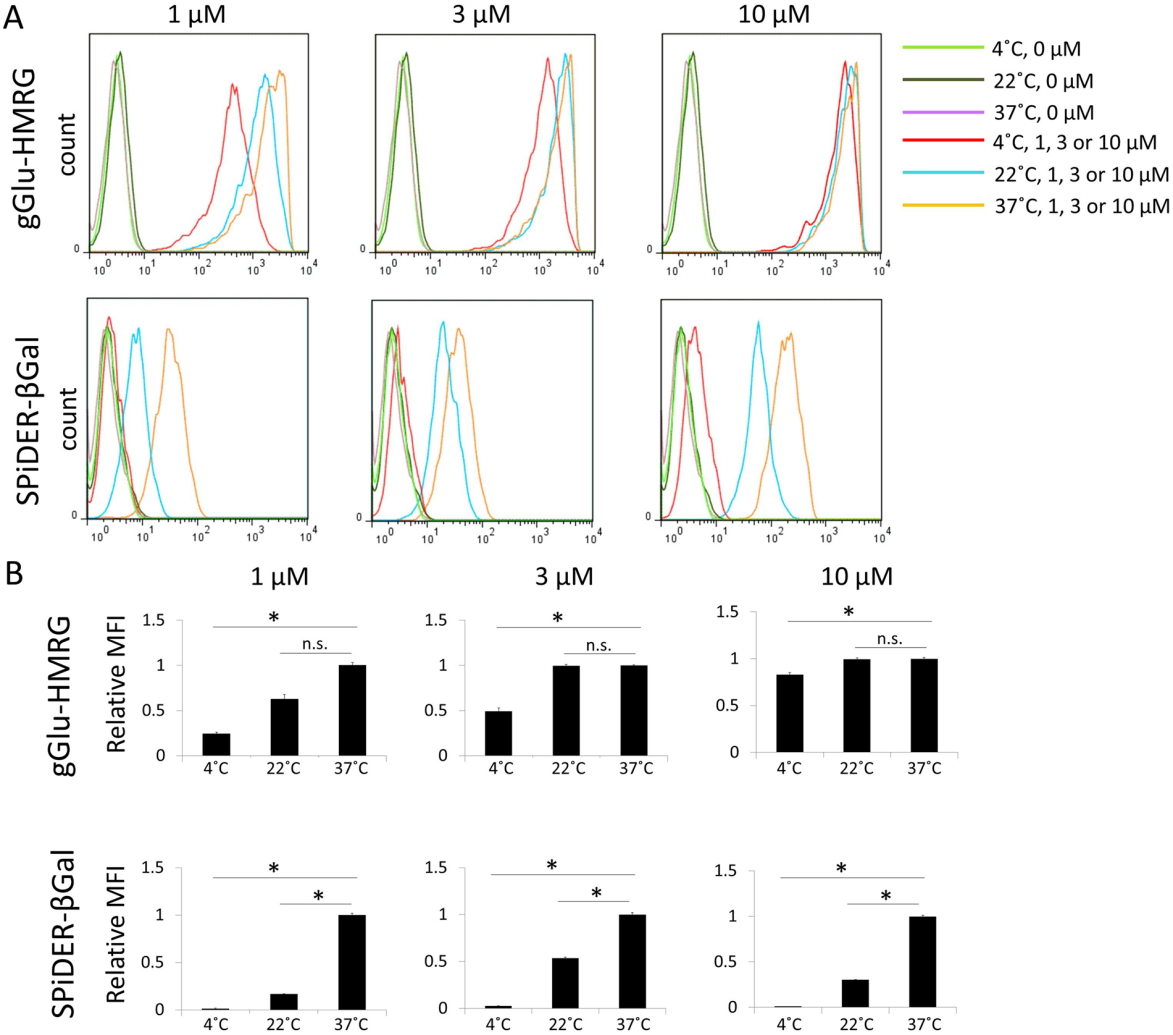
**(A)** Flow cytometric analysis. One representative individual is shown. **(B)** Relative MFI of gGlu-HMRG and SPiDER-βGal in SHIN3 cells. Asterisks show statistical significance of relative MFI between different temperatures. (n = 6 in each group).

Flow cytometry analysis showed consistent results with the microscopic analysis. The gGlu-HMRG group showed statistically significantly higher fluorescence signals compared to SPiDER-βGal at all concentrations or temperatures. gGlu-HMRG was activated and detected even at 4°C, whereas fluorescence signal of SPiDER-βGal was not detected at 4°C (Figure 3A). The relative mean fluorescence intensity (MFI) of the gGlu-HMRG group was also statistically higher compared to that of the SPiDER-βGal group regardless of concentration (p < 0.05). Additionally, there was no statistically significant difference between fluorescence at 22°C and 37°C in the gGlu-HMRG group (Figure 3B).

### *Ex vivo* activatable imaging of fresh and frozen tumors

**Figure 4:**
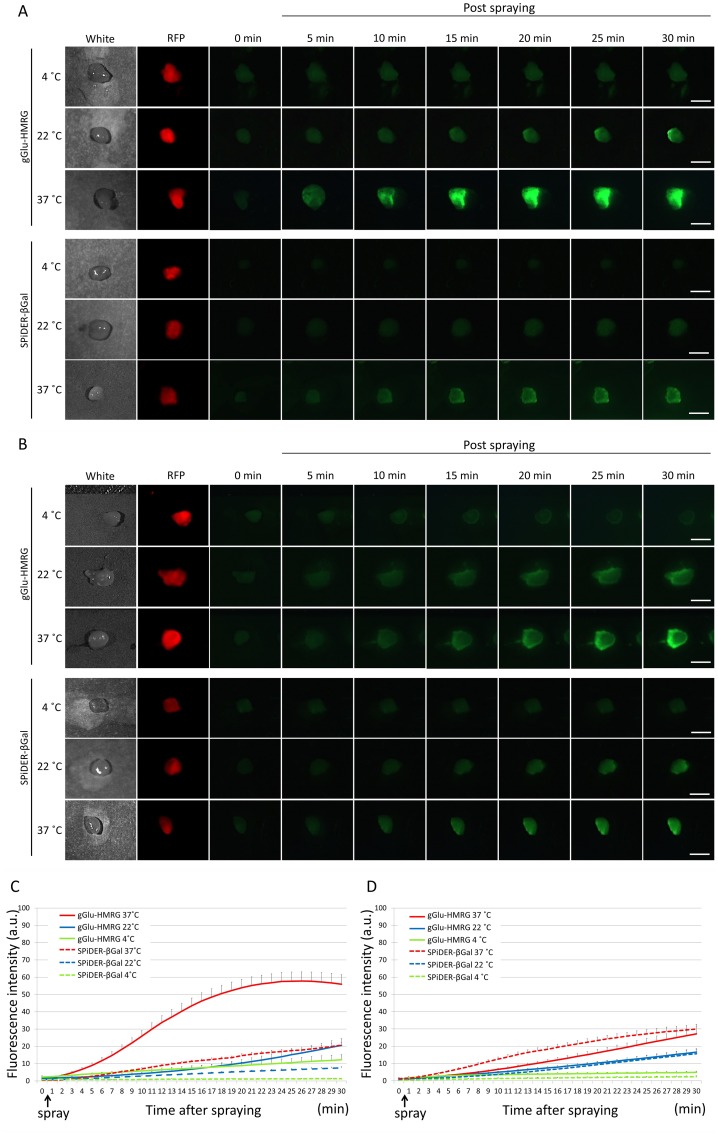
Fluorescence images after the probe is sprayed on the extracted tumor Sequential fluorescence for 30 min, starting from just after spraying with 10 μM gGlu-HMRG and 50 μM SPiDER-βGal at 4°C, 22°C, and 37°C in fresh specimens **(A)** and frozen specimens **(B)**. Time fluorescence intensity of the extracted tumors after spraying for fresh tumors **(C)** and frozen then thawed tumors **(D)** are shown. Data are mean fluorescence intensities ± SEM of tumors at different time points (n = 9 in each group). Bars are 5 mm.

The effect of temperature on fluorescence activation of both probes in fresh and frozen specimens was examined and quantitatively assessed on acquired images (Figure 4). In the fresh specimens after gGlu-HMRG or SPiDER-βGal was sprayed on the tissue, higher temperature yielded stronger fluorescence signal in both groups. The fluorescence intensity at 22°C in the gGlu-HMRG group was similar to that obtained at 37°C in the SPiDER-βGal group. The fluorescence intensity at 4°C with gGlu-HMRG and that at 4°C and 22°C with SPiDER-βGal was so low that tumor margins were invisible during the entire observation period (Figure 4A and 4C). In frozen specimens, both probes at 37°C showed clear margins within 20 minutes. The specimens with SPiDER-βGal at 22°C or with both probes at 4°C showed low fluorescence signals and unclear margins even at 30 minutes (Figure 4B and 4D).

## DISCUSSION

Enzymes are proteins that act as catalysts in biological and pathological processes. Activity of enzymes is generally altered by temperature, which was seen in *in vitro* studies [27]. Hydrolytic activity of GGT and β-galactosidase is maximal at around 37°C or body temperature, then gradually decreases as temperature decreases [28]. Since the temperature of resected tissues quickly falls after removal from the body, investigating the temperature dependence of these probes is critical to optimizing their use for detecting tumor margins. After the application of gGlu-HMRG the extracted specimens showed high fluorescence signals and clear margins even at 22°C that was comparable to the results with SPiDER-βGal at 37°C. The activity of β-galactosidase at low temperature is known to be compromised [29, 30], therefore, it was not surprising that the fluorescence signal of SPiDER-βGal was insufficient to produce visible margins even at room temperature. Therefore, *ex vivo* imaging of resected specimens at room temperature is possible for gGlu-HMRG, but successful use of SPiDER-βGal requires that the specimens be heated close to body temperature.

We observed that freshly-extracted specimens incubated with gGlu-HMRG showed significant fluorescence intensity even at 22°C, suggesting that the tumor margin could be easily detected in the operation room. SPiDER-βgal at 37°C was comparable to that of gGlu-HMRG at 22°C for both fresh and thawed specimens, suggesting that the use of SPiDER-βGal requires the tissue to be brought to a temperature of 37°C. Compared to fresh specimens, thawed specimens showed low fluorescence intensity after the topical application of gGlu-HMRG. After reacting with GGT on the cell surface, gGlu-HMRG releases HMRG, which is the activated and fluorescent form of the probe, which internalizes into cells using both active and passive transport and eventually accumulating in the lysosome due to its acidic pH condition. The process of freezing and thawing reduces active transport functions and may increase pH in the lysosome that could compromise the accumulation of HMRG in the lysosome. On the other hand, frozen then thawed specimens sprayed with SPiDER-βGal showed comparable or even higher fluorescence signal than fresh specimens. This may be due to freeze-thaw induced membrane damage with increased permeability and stabilization of the activated form SPiDER-βGal to intracellular proteins [10]. With either probe, although frozen tissue can be thawed and analyzed, temperature control is even more crucial than with fresh specimens. Taken together, a probe which is trapped in cells by a chemical linkage such as SPiDER-βGal yields similar fluorescence signal regardless of freshness of tissue samples. In contrast, fluorescence signal intensity of a probe which is trapped in cells depending on physiological pH such as gGlu-HMRG greatly relies on freshness of tissue samples.

In conclusion, although imaging of fresh samples at 37°C is the best condition for both probes, successful imaging with gGlu-HMRG can be achieved even at room temperature with thawed samples. A sprayable enzyme-activatable fluorescence probe such as gGlu-HMRG will not only be applied for fluorescence-guided surgery *in vivo*, but also supplement IFSA to evaluate the border of invasive cancer on resected *ex vivo* specimens under optimized temperature conditions.

## MATERIALS AND METHODS

### Reagents

gGlu-HMRG, a GGT-activated fluorescent probe, was synthesized as described previously [9]. GGT is a cell surface enzyme involved in extracellular glutathione metabolism and drug resistance, as well as in leukotriene catabolism [31, 32]. SPiDER-βGal was obtained from Dojindo Molecular Technologies, Inc. (Rockville, MD, USA) [20]. β-galactosidase catalyzes the hydrolysis of lactose into glucose and galactose.

### Cell lines and culture

An established human ovarian cancer cell line, SHIN3, was used for *in vitro* fluorescence microscopy and flow cytometry. High expression of GGT and high expression of β-galactosidase have been reported in SHIN3 [9, 19]. SHIN3 cells were transfected with a plasmid expressing red fluorescent protein (RFP) to create a red fluorescent phenotype (SHIN3-RFP), as previously described [33] and were used for *ex vivo* tumor imaging. Cell lines were grown in RPMI 1640 medium (Life Technologies, Gaithersburg, MD, USA) supplemented with 10 % fetal bovine serum (FBS) (Life Technologies) and 1 % penicillin-streptomycin (Life Technologies) in tissue culture flasks in a humidified incubator at 37°C in 5 % carbon dioxide.

### Fluorescence microscopy analysis

1 × 10^5^ cells from each cell line were plated on a culture well covered by a glass-cover slip and incubated in culture medium for 24 h. Both temperature and probe concentration were tested for fluorescence intensity. gGlu-HMRG or SPiDER-βGal (1 *μ*M, 3*μ*M and 10*μ*M) was added to the culture medium and incubated for 30 min at three temperature variations, 4°C, 22°C (room temperature) or 37°C. After incubation, cells were washed once with phosphate buffered saline (PBS). Fluorescence microscopy was performed using an Olympus BX61 microscope (Olympus America, Inc., Melville, NY) equipped with the following filters: excitation wavelength range 450–490 nm and emission wavelength range 500–550 nm. Transmitted light differential interference contrast (DIC) images were also acquired.

### Flow cytometric analysis

SHIN3 cells (1 × 10^6^) were plated in a 24-chamber culture well and incubated for 24 h. After 1 µM, 3 µM, or 10 µM of gGlu-HMRG or SPiDER-bGal was added to the culture medium, cells were incubated for 30 min at three different temperature conditions: 4°C, 22°C (room temperature) or 37°C. After incubation, cells were washed once with PBS, quickly scraped, and suspended in FACS buffer (PBS supplemented with 5% FBS) on ice. Propidium iodide (PI)-positive dead cells were gated out and the remaining cells were analyzed using a FACS Calibur (BD BioSciences, San Jose, CA, USA). Relative MFI was quantified as the ratio of MFI at each temperature to MFI at 37°C using CellQuest software (BD BioScience, San Jose, USA).

### Tumor model

All *in vivo* procedures were conducted in compliance with the Guide for the Care and Use of Laboratory Animal Resources (1996), US National Research Council, and approved by the local Animal Care and Use Committee. Eight-week-old female homozygote athymic nude mice were purchased from Charles River (NCI, Frederick, MD). SHIN3-RFP cells (2 × 10^6^) suspended in 200 µL of PBS was subcutaneously injected in the right and left dorsi of mice. Experiments with tumor-bearing mice were performed 14 days after injection of the cells.

### *Ex vivo* fluorescence imaging of fresh and frozen tumors

Mice with tumors were euthanized by carbon dioxide inhalation and the subcutaneous tumors were immediately extracted. In order to examine the effect of temperature on enzyme activity, the probe was sprayed on fresh SHIN3-RFP tumors at three different temperatures, 4°C, 22°C and 37°C. Furthermore, another set of tumors was temporarily frozen in liquid nitrogen and thawed at 4°C, 22°C and 37°C. Diluted aqueous solutions (300 μL) of gGlu-HMRG (10 μM) or SPiDER-βGal (50 μM) in PBS were dropped on the extracted tumor (n = 9 in each group). Imaging for the group at 4°C was performed on ice and the group at 37°C was on a heating pad. The temperature of specimens was confirmed by a thermal camera (FLIR C2, FLIR Systems, Inc., Wilsonville, OR, USA) before spraying. Serial fluorescence imaging was recorded every minute for 30 min using a portable fluorescence camera (Discovery INDEC BioSystems, Santa Clara, CA, USA) [34] with the following filter set: band-pass filter from 450 to 490 nm for excitation light and from 511 to 551 nm for emission light, with an exposure time of 50 msec. RFP images were used as a reference for optical identification of SHIN3 cells with the Maestro *In-Vivo* Imaging System (Cri, Woburn, MA, USA). The following filter set was used: a band-path filter from 503 to 555 nm for excitation light and a long-pass filter over 645 nm for emission light. The tunable emission filter was automatically stepped in 10 nm increments from 600 to 800 nm at constant exposure times. The spectral fluorescence images consisting of spectra from autofluorescence and RFP were then unmixed, based on their known spectral patterns using commercial software (Maestro software; CRi). Regions of interest (ROIs) were drawn within the tumor nodules depicted by the RFP images and then the average fluorescence intensity of each ROI was measured. Fluorescence intensity ratio was calculated from the average fluorescence intensity at each time point divided by that at baseline. All fluorescence images were analyzed with ImageJ software (http://rsb.info.nih.gov/ij/).

### Statistical analysis

Comparisons between two groups were made using Student’s t-test (2-tailed). Differences among more than two groups were analyzed using one-way ANOVA followed by post hoc Bonferroni (3 groups) or Tukey-Kramer (>3 groups) tests. Values of p less than 0.05 were considered significant. Error bars represent standard error of the mean (SEM).

## Abbreviations

gGlu-HMRG: γ-glutamyl hydroxymethyl rhodamine green
CT: computed tomography
MRI: magnetic resonance imaging
MMP-2: matrix metalloproteinases-2
β-Gal: β-galactosidase
GGT: γ-glutamyltransferase
IFSA: intraoperative frozen section analysis
RFP: red fluorescent protein
PBS: phosphate buffered saline
DIC: differential interference contrast
PI: propidium iodide
MFI: mean fluorescence intensity
ROI: region of interest
SEM: standard error of the mean
